# Perceived Obstacles Faced by Diabetes Patients Attending University of Gondar Hospital, Northwest Ethiopia

**DOI:** 10.3389/fpubh.2018.00081

**Published:** 2018-03-27

**Authors:** Akshaya Srikanth Bhagavathula, Eyob Alemayehu Gebreyohannes, Tadesse Melaku Abegaz, Tamrat Befekadu Abebe

**Affiliations:** ^1^Department of Clinical Pharmacy, School of Pharmacy, University of Gondar-College of Medicine and Health Sciences, Gondar, Ethiopia; ^2^Department of Learning, Informatics, Management and Ethics (LIME), Karolinska Institutet, Solna, Sweden

**Keywords:** diabetes mellitus, self-care, diabetes obstacles, Gondar, Ethiopia

## Abstract

**Background:**

Diabetes mellitus (DM) is a non-communicable, chronic, and progressive disease that can lead to serious complications and even to premature death. A closer understanding of the DM patients’ specific obstacles will provide a greater clarity of the factors influencing their disease-related quality of life and coping with daily life. The study aimed to evaluate the obstacles of DM patients attending ambulatory clinic of the University of Gondar Hospital (UOGH), Northwest Ethiopia.

**Methods:**

A cross-sectional study was conducted from February to April 2017 at ambulatory clinic of the UOGH. A validated short version of the diabetic obstacle questionnaire was used. The internal reliability of the questionnaire was checked using Cronbach’s alpha and was found to be 92.5%. To determine any association between each of the nine sections of the questionnaire and age, sex, residence, educational status, and DM type, a binary logistic regression was performed.

**Results:**

The mean age of respondents was 38.69 ± 15.39 years. Compared with patients with type 1 DM, patients with type 2 DM reported poorer relationships with medical professionals (adjusted odds ratio (AOR): 2.191, *p*-value = 0.027) and less support from families and friends (AOR: 1.913, *p*-value = 0.049). Patients coming from rural areas (AOR: 2.947, *p* = 0.002) and having no formal education (AOR: 2.078, *p* = 0.029) also received less support from families and friends.

**Conclusion:**

DM patients in UOGH reported several obstacles related to patients’ relationship with health professionals, lack of support from their friends, lack of knowledge about DM, and lack of motivation to exercise. Effective efforts should be initiated to improve healthier environment to educate, care and preventive services for people with DM.

## Introduction

Diabetes mellitus (DM) is a non-communicable, chronic, and progressive disease that can lead to serious complications and even to premature death. The 2014 global estimates showed that 422 million adults were living with DM. It is no longer a disease of only developed nations and has been increasingly affecting developing countries as well (1). In sub-Saharan Africa (SSA), an estimated 14.2 (9.5–29.4) million people aged 20–79 have diabetes, a figure that is expected to increase to 34.2 million by 2040 (2).

Diabetes mellitus is known to influence the patients’ physical, social, and psychological well-being (3). Poor diet, lack of physical activities, and poor self-monitoring of glucose levels are some of the most common obstacles faced by DM patients (3–6). Better glycemic control can progressively prevent or delay complications and improve quality of life (QOL). On the other hand, lack of attention, insufficient time to provide patient-specific education, and overcrowding of health institutions by DM patients are some of the perceived translational obstacles faced by the healthcare providers (7). A closer understanding of the DM patients’ specific obstacles will provide a greater clarity of the factors influencing their disease-related QOL and coping with daily life.

Several studies were conducted using different diabetes-specific QOL instruments conducted on target population (type 1 or type 2 or both) among different ethnic communities and countries (8). However, popular scales such diabetes-related QOL, the Audit of Diabetes-Dependent Quality of Life (ADDQOL), and Problem Areas in Diabetes (PAID) have been widely used across different populations. In contrast, the Diabetes Health Profile (DHP), Questionnaire on Stress in Diabetes Patients-Revised (QSD-R), and Diabetes Quality of Life Clinical Trials Questionnaire (DQLCT-R) have not been used in any studies (8). All these scales frequently measured health-related QOL (HRQOL) and little attention was given to understand the patient-specific obstacles. Furthermore, considering selection of Africans, only few studies assessed adherence (9), mental health outcomes (10), and two randomized controlled trials (11, 12) conducted on African-American communities with DM. In the context of DM patients in SSA, no studies were identified investigating DM patients-specific obstacles in Ethiopia. Therefore, the study aimed to evaluate the obstacles of DM patients attending ambulatory clinic of the University of Gondar Hospital (UOGH), Northwest Ethiopia.

## Materials and Methods

### Study Design and Setting (Area and Period)

A cross-sectional study was conducted from February to April 2017 at the ambulatory clinic of the UOGH. UOGH is located in the north-west part of Ethiopia 727 km from the capital city Addis Ababa.

#### Operational Definition

- Type 1 DM: also known as insulin-dependent diabetes mellitus (IDDM) caused by β-cell failure and severe or absolute insulin deficiency (13).
- Type 2 DM: also known as non-insulin dependent diabetes mellitus (NIDDM) is a complex multisystem disease with carbohydrate and lipid metabolic derangements, characterized by vascular inflammation. All characterized by elevated glycemic markers caused by a secretory defect of insulin on the background of insulin resistance (13)
- Formal education: having at least primary-school education
- Rural areas: a residential area located outside the towns and cities

#### Sample Size

An online sample size calculator "Creative research systems" (14) was used to determine the number of DM participants for the study, by considering 95% confidential level, with an accuracy of 50% for the population of 2,125 registered DM patients at the outpatient department of the UOGH (15) has given a confidential level of 6.42, recommended a sample size of 210.

### Study Population

Type 1 and type 2 diabetic patients attending ambulatory clinic during the study period were recruited and included in the study. However, patients with cognitive impairment, severely sick, and who did not consent to participate in the study were excluded.

### Study Instrument

The validated short version of the diabetic obstacle questionnaire (DOQ-30) was developed by researchers and was validated in six European countries (6). Permission was obtained to reuse from the corresponding author. The questionnaire consisted of a total of 30 questions that were divided in to nine sections addressing different issues: relationships with medical professionals (four items), support from friends and families (four items), knowledge of the disease (four items), lifestyle changes (four items), self-monitoring (four items), uncertainty about a consultation (two items), medication (two items), and insulin use (two items). A 4-point Likert scale (1 = Strongly disagree, 2 = Disagree, 3 = Agree, and 4 = Strongly agree) was used to obtain their responses. Before the questionnaire was administered to patients, it was first translated into Amharic by two of the investigators (EAG and TMA) and then back-translated to English by another investigator (TBA) to verify for accuracy. The translated questionnaire was pretested among 20 randomly selected DM patients for reliability and validity. No language problems and corrections were requested. The questionnaire was basically self-administered; however, in cases where the patients were not able to read and write interview method was employed. Sufficient time was given for the patients to fill the questionnaire during their waiting time. Any discrepancies were dealt with discussion. The internal reliability of the questionnaire was checked using Cronbach’s alpha and was found to be 92.5%.

### Ethical Consideration

Ethical clearance was obtained from the ethical clearance committee of the school of pharmacy. Before the questionnaire was administered to patients, the aim of the study was explained to patients, and written and verbal informed consent was obtained from each patient prior to their participation. The information obtained from patients was kept confidential and was used for the study purpose only. All the patients in the study were voluntary and no compensation was provided. Furthermore, confidentiality was maintained by not disclosing any personal information of the study participants and their responses were randomized.

### Statistical Analysis

Descriptive statistics were used to summarize sociodemographic characteristics and ratings of the different components of the questionnaire by the study participants. To determine any association between each of the nine sections of the DOQ-30 and age, sex, residence, educational status, and DM type a binary logistic regression was performed. To do so, an average score of the items (1 to 4 in the Likert scale) in each of the eight sections was made. Then the nine sections were dichotomized into two: Strongly disagree/Disagree if the average score was ≤2 and Agree/Strongly Agree if the average score was >2.

## Results

### Baseline Characteristics of Respondents

Of 210 patient approached, 202 subjects responded to the questionnaire, giving a 96.2% response rate. The mean ± SD age of respondents was 38.69 ± 15.39. Majority of the study participants were male (61.9%) and came from urban areas 117 (57.9%) (Table 1).

**Table 1:** Demographic characteristics of diabetic patients in University of Gondar Hospital (*N* = 202).

Characteristics	Frequency (%)
Mean age ± SD	38.69 ± 15.393
**Sex**	
Male	125 (61.9%)
Female	77 (38.1)
**Residence**	
Urban	117 (57.9)
Rural	85 (42.1)
**Marital status**	
Single	63 (31.2)
Married	113 (55.9)
Divorced	21 (10.4)
Widowed	5 (2.5)
**Education level**	
No formal education	85 (42.1)
Primary	47 (23.3)
Secondary	33 (16.3)
College and above	37 (18.3)
**Occupation**	
None	3 (1.5)
Student	39 (19.3)
Merchant	30 (14.9)
Government employee	39 (19.3)
Farmer	84 (41.6)
Daily laborer	7 (3.5)
**Monthly income (Ethiopian Birr)**	
Less than 500	115 (56.9)
500–999	11 (5.4)
1,000–1,999	35 (17.3)
2,000–3,000	41 (20.3)

Majority of the patients (57.4%) were having type 1 DM. Nearly two-third of patients (67.3%) were taking a single anti-diabetic agent of which insulin was the most commonly used one (57.9%). The remaining patients were using a combination of antidiabetic medications (Table 2).

**Table 2:** Disease and treatment characteristics of diabetic patients in University of Gondar Hospital (*N* = 202).

Characteristics	Frequency (%)
**Type of diabetes**	
Type 1	116 (57.4)
Type 2	86 (42.6)
**Number of antidiabetic medications used**	
1medication	136 (67.3)
2medications	66 (32.7)
**Current antidiabetic regimen**	
Insulin	117 (57.9)
Metformin	17 (8.4)
Insulin plus metformin	12 (5.9)
Insulin plus glibenclamide	17 (8.4)
Metformin plus glibenclamide	38 (18.8)
**Outcome**	
Improved	155(76.8)
Not improved	47(23.2)

#### Relationship With Health Professionals and Support From Friends and Family

With the exception of the “I am not assisted in setting realistic targets for changing my lifestyle.” item, nearly two-third of the respondents reported that they have a poor relationship with health professionals. Majority of the participants reported getting poor social support from their friends (53.5%) and feeling lonely with their disease (51.5%), but received but good social support from their family (53.3%) and showed good attitude to manage DM had they had support from others (54.5%) (Table 3).

**Table 3:** Rating of patients for individual components of diabetes obstacles.

Obstacles	Mean ± SD	Agree/totally agree *n*(%)	Disagree/totally disagree *n*(%)
**Relationships with medical professionals**			
1. I am not assisted in setting realistic targets for changing my lifestyle	2.94 (1.526)	84 (41.6)	118 (58.4)
2. Treatment alternatives are not explained to me	3.63 (1.481)	128 (63.4)	74 (36.6)
3. I have not been told what to expect from my treatment	3.7 (1.443)	131 (64.9)	71 (35.1)
4. The good and bad aspects of each choice have not been discussed with me	3.82 (1.463)	136 (67.3)	66 (32.7)

**Support from friends and family**			
5. I feel I get little support from my family	3 (1.71)	94 (46.5)	108 (53.5)
6. I feel I get little support from my friends	3.24 (1.598)	108 (53.5)	94 (46.5)
7. I feel very alone with my diabetes	3.15 (1.509)	104 (51.5)	98 (48.5)
8. I would manage my diabetes much better if I had encouragement socially	3.24 (1.494)	110 (54.5)	92 (45.5)

**Knowledge of the disease**			
9. I have difficulty understanding the information from literature	3.39 (1.532)	114 (56.4)	88 (43.6)
10. I do not know as much as I need to know to manage my diabetes	3.35 (1.51)	108 (53.5)	94 (46.5)
11. I do not know as much as I need to know about the consequences of having diabetes	3.58 (1.441)	120 (59.4)	82 (40.6)
12. I do not know enough about the treatment for diabetes	3.41 (1.501)	117 (57.9)	85 (42.1)

**Lifestyle changes**			
13. My diabetes has placed a strain on my personal relationships	3.18 (1.441)	99 (49.09)	103 (51.0)
14. Changes in my diet have put a strain on my family	3 (1.454)	91 (45.0)	111 (55.0)
15. I feel resentful that I am obliged to change my eating habits	3.1 (1.373)	94 (46.5)	108 (53.5)
16. My diabetic diet spoils my social life	3.19 (1.465)	100 (49.5)	102 (50.5)

**Exercising**			
17. I have not found an exercise I enjoy	3.25 (1.289)	102 (50.5)	100 (49.5)
18. I lack the motivation to exercise	3.47 (1.294)	117 (57.9)	85 (42.1)
19. I am unable to fit exercise into my lifestyle	3.47 (1.309)	116 (57.4)	86 (42.6)
20. I am unable to afford the cost of exercising on a regular basis	3.21 (1.378)	103 (51)	99 (49)

**Self-monitoring**			
21. Self-monitoring makes me feel frustrated	3.05 (1.445)	90 (44.6)	112 (55.4)
22. I find it too uncomfortable to self-monitor	2.92 (1.417)	81 (40.1)	121 (59.9)
23. I find it especially hard to test when I am busy	2.92 (1.401)	85 (42.1)	117 (57.9)
24. Self-monitoring makes me fearful of a high reading	2.95 (1.422)	82 (40.6)	120 (59.4)

**Uncertainty about a consultation**			
25. I feel a sense of helpless when consulting with nurses	2.95 (1.411)	79 (39.1)	123 (60.9)
26. The way that I was told that I had diabetes made feel afraid	2.93 (1.475)	82 (40.6)	120 (59.4)

**Medication**			
27. I do not feel I am being prescribed a medication dose that is right for me	3.01 (1.461)	88 (43.6)	114 (56.4)
28. I do not feel I am being prescribed medication that is right for me	2.72 (1.457)	68 (33.7)	134 (66.3)

**Insulin use**			
29. Using insulin makes life too complicated	2.92 (1.465)	87 (43.1)	115 (56.9)
30. Using insulin means my diabetes is getting worse	3.06 (1.503)	95 (47.0)	107 (53.0)

#### Knowledge About Diabetes, Lifestyle Changes, and Exercise

The greater number of DM patients reported having poor knowledge about the disease in terms of understanding information from literature (56.4%), managing their diabetes (53.5%), consequences of diabetes (59.4%), and treatment for diabetes (57.9%). On the other hand, less than half of the respondents reported having difficulty with lifestyle changes while most of the respondents reported that they faced different challenges to do regular physical exercise (Table 3).

#### Medication Appropriateness and Insulin Use

More than half of respondents (56.4%) agreed that they received the right dose while two-thirds of them (66.3%) stated that the type of antidiabetic agent was appropriate for their disease. In addition, most of the patients reported that insulin use neither complicates their life (56.9%) nor is a sign of worsening of their diabetes (53.0%) (Table 3).

#### Self-Monitoring

Self-monitoring was not considered a challenge by most of the patients. Less than half of the respondents reported that self-monitoring made them frustrated (44.6%) and fearful of a high reading (40.6%), became uncomfortable to self-monitor (40.1%), and found it hard to test when busy (42.1%) (Table 3).

Binary logistic regression identified few variables as independent predictors of higher frequency of obstacles among diabetic patients. Compared with patients with type 1 DM, patients with type 2 DM reported poorer relationships with medical professionals [AOR (95% CI): 2.191 (1.092–4.399), *p*-value = 0.027] and less support from families and friends [AOR (95% CI) = 1.913 (1.002–3.653), *p*-value = 0.049]. Patients coming from rural areas [AOR (95% CI): 2.947 (1.485–5.848), *p* = 0,002] and having no formal education [AOR (95% CI): 2.078 (1.08–3.998), *p* = 0.029] also received less support from families and friends. Coming from rural areas was associated with having poorer knowledge [AOR (95% CI): 10.132 (4.492–22.851), *p* = 0.000] and facing difficulties with lifestyle changes [AOR (95% CI): 3.45 (1.792–6.64), *p* = 0.000], exercising [AOR (95% CI): 4.859 (2.279–10.359), *p* = 0.000], self-monitoring [AOR (95% CI): 4.817 (2.551–9.097), *p* = 0.000], and insulin use [AOR (95% CI): 2.082 (1.179–3.675), *p* = 0.011]. Similar obstacles were also reported by patients having no formal education (Table 4).

**Table 4:** Diabetes obstacles and associated factors.

Characteristics	Age	Sex		Residence		Education		DM type	
		Male	Female	Urban	Rural	Formal education	No formal education	Type 1	Type 2
**Relationships with medical professionals**									
AOR (95% CI)	1.016 (0.994–1.039)	1	1.093 (0.561–2.127)	1	1.358 (0.699–2.635)	1	1.484 (0.759–2.899)	1	2.191 (1.092–4.399)
*p*-Value	0.154		0.794		0.366		0.248		**0.027**

**Support from friends and family**									
AOR (95% CI)	1.019 (0.998–1.041)	1	1.198 (0.633–2.268)	1	2.947 (1.485–5.848)	1	2.078 (1.08–3.998)	1	1.913 (1.002–3.653)
*p*-Value	0.074		0.578		**0.002**		**0.029**		**0.049**

**Knowledge of the disease**									
AOR (95% CI)	1.035 (1.013–1.057)	1	1.091 (0.597–1.994)	1	10.132 (4.492–22.851)	1	4.347 (2.205–8.569)	1	1.736 (0.947–3.183)
*p*-Value	**0.002**		0.778		**0.000**		**0.000**		0.074

**Lifestyle changes**									
AOR (95% CI)	1.001 (0.982–1.021)	1	1.2 (0.654–2.199)	1	3.45 (1.792–6.64)	1	2.046 (1.105–3.789)	1	0.831 (0.461–1.497)
*p*-Value	0.885		0.556		**0.000**		**0.023**		0.831

**Exercising**									
AOR (95% CI)	1.015 (0.994–1.036)	1	1.432 (0.747–2.745)	1	4.859 (2.279–10.359)	1	2.24 (1.153–4.354)	1	0.805 (0.433–1.497)
*p*-Value	0.177		0.28		**0.000**		**0.017**		0.493

**Self-monitoring**									
AOR (95% CI)	1.003 (0.985–1.022)	1	1.42 (0.793–2.544)	1	4.817 (2.551–9.097)	1	2.675 (1.475–4.852)	1	0.703 (0.399–1.238)
*p*-Value	0.738		0.238		**0.000**		**0.001**		0.222

**Uncertainty about a consultation**									
AOR (95% CI)	1.002 (0.984–1.02)	1	1.064 (0.603–1.878)	1	1.588 (0.904–2.789)	1	1.875 (1.064–3.305)	1	0.674 (0.385–1.181)
*p*-Value	0.811		0.831		0.108		**0.03**		0.168

**Medications**									
AOR (95% CI)	1.013 (0.995–1.032)	1	1.002 (0.568–1.769)	1	2.521 (1.421–4.474)	1	1.957 (1.11–3.447)	1	0.976 (0.559–1.706)
*p*-Value	0.159		0.994		**0.002**		**0.02**		0.933

**Insulin use**									
AOR (95% CI)	1.026 (1.007–1.045)	1	1.643 (0.927–2.913)	1	2.082 (1.179–3.675)	1	3.204 (1.787–5.745)	1	1.688 (0.962–2.963)
*p*-Value	**0.008**		0.089		**0.011**		**0.000**		0.068

*Significant at P<0.05 were bolded*.

## Discussion

The current research assessed the obstacles faced by both type 1 and type 2 DM patients attending the ambulatory clinic of UOGH. Based on the findings of this study, poor relationship with health professionals, lack of support from their friends, and feeling lonely with their disease were reported as obstacles by more than half of the DM patients. However, poor knowledge about the disease and obstacles to take medications properly were also reported as obstacles by the patients. There were few differences between type 1 and type 2 DM patients in the reported intensity of obstacles. In the present study, type 1 DM patients and type 2 DM patients significantly differed in their relationship with medical professionals, and receiving support from family and friends. Other studies also reported difference in obstacles faced by patients with type 1 and type 2 DM (16, 17).

These data point to three major types of diabetes obstacles: the desire to have a good relationship with the health professionals, and more broadly, willing to take support from their family and friends, and lack of knowledge about DM is making them feel lonely (burden). It is noteworthy that there were relatively few consistent differences in factor scores among patient characteristics (age, sex, and type of DM) pointing to the potential generalizability of the obstacles faced across the DM population.

Lower level of education and living in rural areas were independently associated with poor family and friends support, lack of knowledge about DM, poor lifestyle changes, less-frequent exercising, and poor self-monitoring and medication use process. Furthermore, we note that these factors remained unchanged even after adjusting for their age, sex and type of DM. This suggests that these are main driving obstacles faced by DM patients in our population.

Non-availability of professional care team (specialists, nurses, dietitians, and diabetes educators), poor healthcare infrastructure, a strong culture of hospitality and frequent fasting has nudged the Ethiopian rural societies. Several obstacles were also identified for the poor DM management such as poor self-monitoring of blood glucose, including lower socioeconomic positions, education levels, social class, and living in a high poverty (18). These suggest that patients with DM might be more likely to follow health professionals’ recommendations to improve their DM knowledge and QOL. Most of our study participants agreed that their medical professionals did not provide time to explain treatment alternatives, and good and bad about their care. This explains the time spent by the medical professional to patients is low. Improving communication amongst doctors, nurses, pharmacists and patients can play a central role in improving peer support, understand patient-specific needs, and facilitate compliance, and self-motivation to control diabetes (19–22). Family members and friends can actively support and care the DM patients. Several studies have highlighted the importance of family, friends, and colleagues in improving well-being and self-management of DM (23–26). Most of our study participants opinioned that they receive little support from friends, feel very lonely because of DM and further, they are confident to manage DM if they receive social support. Family members are often asked to share in the responsibility of disease management. Their support can drive patient to fix their appointments, or helping inject insulin, and social and emotional support in helping patients cope with DM (27–29). Engaging family members or friends in diabetes education program can have a greater improvement in increasing knowledge of DM, glycemic control, and provide instrumental and social support than the patients alone.

DM-related knowledge is essential to cope with emotional aspects, glycemic control, and to prevent DM-related complication. Several studies assessed psychosocial outcomes found some improvement in DM patients’ depressive symptoms, DM-related destress, self-efficacy, perceived social support, and DM-related QOL (30–33). But, several studies found no change in their improvement in only some self-care behaviors. Understanding these discrepancies, educating patients along with their family members about diabetes-care needs can help them to understand the importance of lifestyle modifications, exercise routines, and healthy recipes, ultimately alter disease progression.

Most of the DM patients believe that DM should only be treated with oral medications and insulin injections. It is evident from the previous studies that most of the DM patients rarely change their diet or exercise habits after they were diagnosed (34–36). In our study, around 40% of the participants reported that they were not well informed about their medications and feeling that using insulin may worsen their DM. In order to minimize such misconceptions and beliefs, health professionals especially pharmacists’ need to educate patients about their medications to improve their level of medication adherence and consequently therapeutic outcomes.

Our study had few limitations. First, a self-reported method was used to assess DM patients’ obstacles. Therefore, respondents may underestimate or overestimate their DM-related obstacles. More precise estimates of real-life obstacles of patients can be obtained through qualitative methods. However, self-reported assessment of their obstacles is practical and inexpensive. Second, although the study is a single center and the sample size was low, it is not a representative of whole Ethiopian diabetic patients. Therefore, caution should be exercised in generalizing our findings. Third, the study was cross-sectional and the selection method might have created a bias toward of obtained responses from patients who attend to the ambulatory clinic are those who usually care about their health. Finally, it could be broadly argued that a focus on patient obstacles is insufficient.

## Conclusion

Diabetes mellitus patients in UOGH have several obstacles in everyday life. Obstacles related to patients’ relationship with health professionals, lack of support from their friends, lack of knowledge about DM, and lack of motivation to exercise were frequently reported. Effective efforts should be initiated to improve healthier environment to educate, care, and preventive services for people with DM.

## Ethics Statement

This study was carried out in accordance with the recommendations of ethical clearance committee of the school of pharmacy with written informed consent from all subjects. All subjects gave written informed consent in accordance with the Declaration of Helsinki. The protocol was approved by the institutional ethical committee of University of Gondar-School of Pharmacy.

## Author Contributions

All authors listed have made a substantial, direct and intellectual contribution to the work, and approved it for publication.

## Conflict of Interest Statement

The authors report no relationships that could be construed as a conflict of interest.
